# Supplementary material for: Relationships between Cognitive Functioning and Powered Mobility Device Use: A Scoping Review
Source: Int J Environ Res Public Health. 2021 Nov 26;18(23):12467. doi: 10.3390/ijerph182312467 (PMC8657167; doi:10.3390/ijerph182312467)
Supplement: Supplementary file 1 [file ijerph-18-12467-s001.zip › ijerph-1468164-supplementary.pdf]

Influence of cognitive functions on powered mobility device use: A systematic review

Web of science (Clarivate)

1.	"electric wheelchair*" or "motorised wheelchair*" or "motorized wheelchair*" or "power* mobility" or "power* wheelchair*" or scooter*	[TOPIC]
2.	"ability to learn*" OR anticipat* OR attention* OR automatism* OR aware* OR cognition* OR ((abilit* OR control* OR defect* OR deficit* OR disabilit* OR disORder* OR dysfunction* OR function* OR impair* OR performance* OR process* OR rehabilitation OR skill*) NEAR/5 cognitive) OR concentrat* OR conditioning OR "decision making" OR "decision-making" OR ((depth OR distance OR space) NEAR/5 perception*) OR "discrimination learn*" OR ((function* OR syndrome) NEAR/5 dysexecutive) OR emotion* OR ((control* OR dysfunction* OR disorder* OR function*) NEAR/5 executive) OR expectation* OR experienc* OR "intellectual function*" OR memory OR "mental concentration" OR "mental function*" OR ORientation OR perception* OR ((disorder* OR function* OR orientation) NEAR/5 perceptual) OR "problem solving" OR "problem-solving" OR ((discrimination OR learn* OR memory OR navigation* OR orientation* OR perception*) NEAR/5 spatial)	[TOPIC]
3.	1 AND 2	

CINAHL (Ebsco))

1.	"electric wheelchair*" or "motorised wheelchair*" or "motorized wheelchair*" or "power* mobility" or "power* wheelchair*" or scooter*	[TI. AB.]
2.	(MH "Wheelchairs, Powered")	Descriptor
**3.**	**1 or 2**	
4.	"ability to learn*" OR anticipat* OR attention* OR automatism* OR aware* OR cognition* OR ((abilit* OR control* OR defect* OR deficit* OR disabilit* OR disorder* OR dysfunction* OR function* OR impair* OR performance* OR process* OR rehabilitation OR skill*) N5 cognitive) OR concentrat* OR conditioning OR "decision making" OR "decision-making" OR ((depth OR distance OR space) N5 perception*) OR "discrimination learn*" OR ((function* OR syndrome) N5 dysexecutive) OR emotion* OR ((control* OR dysfunction* OR disorder* OR function*) N5 executive) OR expectation* OR experienc* OR "intellectual function*" OR memory OR "mental concentration" OR "mental function*" OR ORientation OR perception* OR ((disorder* OR function* OR orientation) N5 perceptual) OR "problem solving" OR "problem-solving" OR ((discrimination OR learn* OR memory OR navigation* OR orientation* OR perception*) N5 spatial)	[TI. AB.]
5.	(MH "Attention")	Descriptor
6.	(MH "Cognition Disorders")	Descriptor
7.	(MH "Cognition")	Descriptor
8.	(MH "Cognitive Ability (Iowa NOC)")	Descriptor
9.	(MH "Cognitive Orientation (Iowa NOC)")	Descriptor
10.	(MH "Concentration (Iowa NOC)")	Descriptor
11.	(MH "Conditioning (Psychology)")	Descriptor
12.	(MH "Decision Making")	Descriptor
13.	(MH "Depth Perception")	Descriptor
14.	(MH "Emotions")	Descriptor
15.	(MH "Executive Function")	Descriptor
16.	(MH "Life Experiences")	Descriptor
17.	(MH "Memory")	Descriptor
18.	(MH "Mental Processes")	Descriptor
19.	(MH "Orientation")	Descriptor
20.	(MH "Perception")	Descriptor
21.	(MH "Problem Solving")	Descriptor
22.	(MH "Rehabilitation, Cognitive")	Descriptor
23.	(MH "Spatial Perception")	Descriptor
24.	5/23 OR	Descriptor
25.	4 or 24	Descriptor

26.	3 AND 25	Descriptor

Chercheur(s)	1. Alice Pellichero	2. Marie Denise Lavoie
Date	2020/03/02	2020/03/02
Résultats		

Influence of cognitive functions on powered mobility device use: A systematic review

EMBASE (Elsevier)

1.	"electric wheelchair*" or "motorised wheelchair*" or "motorized wheelchair*" or "power* mobility" or "power* wheelchair*" or scooter*	[ti. ab.]
2.	'powered wheelchair'/de	Emtree
3.	**1 or 2**	
4.	"ability to learn*" OR anticipat* OR attention* OR automatism* OR aware* OR cognition* OR ((abilit* OR control* OR defect* OR deficit* OR disabilit* OR disORder* OR dysfunction* OR function* OR impair* OR performance* OR process* OR rehabilitation OR skill*) NEAR/5 cognitive) OR concentrat* OR conditioning OR "decision making" OR "decision-making" OR ((depth OR distance OR space) NEAR/5 perception*) OR "discrimination learn*" OR ((function* OR syndrome) NEAR/5 dysexecutive) OR emotion* OR ((control* OR dysfunction* OR disorder* OR function*) NEAR/5 executive) OR expectation* OR experienc* OR "intellectual function*" OR memory OR "mental concentration" OR "mental function*" OR orientation OR perception* OR ((disorder* OR function* OR orientation) NEAR/5 perceptual) OR "problem solving" OR "problem-solving" OR ((discrimination OR learn* OR memory OR navigation* OR orientation* OR perception*) NEAR/5 spatial)	
**5.**	'cognition'/de	Emtree
6.	'anticipation'/de	Emtree
7.	'attention'/de	Emtree
8.	'awareness'/de	Emtree
9.	'automatism'/de	Emtree
10.	'cognitive defect'/de	Emtree
11.	'cognitive rehabilitation'/de	Emtree
12.	'conditioning'/de	Emtree
13.	'decision making'/de	Emtree
14.	'depth perception'/de	Emtree
15.	'discrimination learning'/de	Emtree
16.	'distance perception'/de	Emtree
17.	'emotion'/de	
18.	'executive function'/de	Emtree
19.	'memory'/exp	Emtree
20.	'mental concentration'/de	Emtree
21.	'mental capacity'/de	
22.	'mental function'/de	
23.	'orientation'/de	Emtree
24.	'perception'/de	Emtree
25.	'personal experience'/de	
26.	'problem solving'/de	Emtree
27.	'spatial discrimination'/de	Emtree

28.	'spatial orientation'/de	Emtree
29.	'spatial learning'/de	Emtree
30.	'spatial memory'/de	Emtree
31.	5/30 or	
32.	4 or 31	
33.	3 AND 32	

Chercheur(s)	1. Alice Pellichero	2. Marie Denise Lavoie
Date	2020/03/02	2019/02/18
Résultats	68	1005

medline (ovid)

1.	"electric wheelchair*" or "motorised wheelchair*" or "motorized wheelchair*" or "power* mobility" or "power* wheelchair*" or scooter*	[ti. ab.]
2.	Wheelchairs/	MESH
**3.**	**1 or 2**	
4.	"ability to learn*" or anticipat* or attention* or automatism* or aware* or cognition* or ((abilit* or control* or defect* or deficit* or disabilit* or disorder* or dysfunction* or function* or impair* or performance* or process* or rehabilitation or skill*) adj5 cognitive) or concentrat* or conditioning or "decision making" or "decision-making" **or (**(depth or distance or space) adj5 perception*) or "discrimination learn*" or ((function* or syndrome) adj5 dysexecutive) or emotion* or ((control* or dysfunction* or disorder* or function*) adj5 executive) or expectation* or experienc* or "intellectual function*" or memory or "mental concentration" or "mental function*" or orientation or perception* or ((disorder* or function* or orientation) adj5 perceptual) or "problem solving" or "problem-solving" or ((discrimination or learn* or memory or navigation* or orientation* or perception*) adj5 spatial)	[ti. ab.]
5.	anticipation, psychological/	MESH
6.	attention/	MESH
7.	automatism/	MESH
8.	awareness/	MESH
9.	cognition disorders/	MESH
10.	cognition/	MESH
11.	cognitive dysfunction/	MESH
12.	"conditioning (psychology)"/	MESH
13.	decision making/	MESH
14.	depth perception/	MESH
15.	discrimination learning/	MESH
16.	distance perception/	MESH
17.	emotions/	MESH
18.	executive function/	MESH
19.	Intelligence/	MESH
20.	memory/	MESH
21.	mental processes/	MESH
22.	orientation/	MESH
23.	orientation, spatial/	MESH
24.	perception/	MESH
25.	problem solving/	MESH
26.	space perception/	MESH

27.	spatial learning/	MESH
28.	spatial memory/	MESH
29.	spatial navigation/	MESH
30.	5/29 or	MESH
**31.**	4 or 30	
32.	3 AND 31	

Chercheur(s)	1. Alice Pallichero	2. Marie Denise Lavoie
Date	2019/02/	2019/02/
Résultats		

PsycInfo (ovid)

1.	"electric wheelchair*" or "motorised wheelchair*" or "motorized wheelchair*" or "power* mobility" or "power* wheelchair*" or scooter*	[ti. ab.]
2.	mobility aids/	Thesaurus
**3.**	**1 or 2**	
4.	"ability to learn*" or anticipat* or attention* or automatism* or aware* or cognition* or ((abilit* or control* or defect* or deficit* or disabilit* or disorder* or dysfunction* or function* or impair* or performance* or process* or rehabilitation or skill*) adj5 cognitive) or concentrat* or conditioning or "decision making" or "decision-making" **or (**(depth or distance or space) adj5 perception*) or "discrimination learn*" or ((function* or syndrome) adj5 dysexecutive) or emotion* or ((control* or dysfunction* or disorder* or function*) adj5 executive) or expectation* or experienc* or "intellectual function*" or memory or "mental concentration" or "mental function*" or orientation or perception* or ((disorder* or function* or orientation) adj5 perceptual) or "problem solving" or "problem-solving" or ((discrimination or learn* or memory or navigation* or orientation* or perception*) adj5 spatial)	[ti. ab.]
5.	attention/ cw Psycinfo classification word (index)	Thesaurus APA
6.	automatism/	Thesaurus APA
7.	awareness/	Thesaurus APA
8.	cognition/	Thesaurus APA
9.	cognitive ability/	Thesaurus APA
10.	cognitive control/	Thesaurus APA
11.	cognitive impairment/	Thesaurus APA
12.	cognitive processes/	Thesaurus APA
13.	cognitive rehabilitation/	Thesaurus APA
14.	concentration/	Thesaurus APA
15.	conditioning/	Thesaurus APA
16.	decision making/	Thesaurus APA
17.	depth perception/	Thesaurus APA
18.	discrimination learning/	Thesaurus APA
19.	distance perception/	Thesaurus APA
20.	dysexecutive syndrome/	Thesaurus APA
21.	emotions/	Thesaurus APA
22.	executive function/	Thesaurus APA
23.	expectations/ (anticipation)	Thesaurus APA
24.	Intelligence/	Thesaurus APA
25.	Life experiences/	Thesaurus APA
26.	learning ability/	Thesaurus APA
**27.**	memory/	Thesaurus APA
28.	perception/	Thesaurus APA
**29.**	perceptual orientation/	Thesaurus APA
30.	problem solving/	Thesaurus APA
31.	spatial learning/	Thesaurus APA
32.	spatial memory/	Thesaurus APA
33.	spatial perception/	Thesaurus APA
34.	"spatial orientation (perception)"/	Thesaurus APA
35.	5/34 or	Thesaurus APA

**36.**	**4 or 35**	
**37.**	**3 AND 36**	

Chercheur(s)	1. Alice Pallichero	2. Marie Denise Lavoie
Date	2019/02/	2019/02/
Résultats		
